# Development of *Trichosomoides nasalis* (Nematoda: Trichinelloidea) in the murid host: evidence for larval growth in striated muscle fibres

**DOI:** 10.1051/parasite/2012191019

**Published:** 2012-02-15

**Authors:** E.H. Fall, M. Diagne, K. Junker, J.M. Duplantier, K. Ba, I. Vallée, O. Bain

**Affiliations:** 1 Département de Biologie Animale, Faculté des Sciences et Techniques, Université Cheikh Anta Diop de Dakar BP5005 Dakar Sénégal; 2 ARC-Onderstepoort Veterinary Institute Private Bag X05 Onderstepoort 0110 South Africa; 3 Centre de Biologie et de Gestion des Populations (UMR INRA/ IRD/CIRAD/Montpellier Supagro), Campus de Baillarguet, CS 30016 34988 Montferrier sur Lez Cedex France; 4 IRD, UR 022 CBGP-Bel Air BP 1386 Dakar CP 18524 Sénégal; 5 UMR BIPAR, Anses, ENVA, UPEC, Laboratory for Animal Health, Anses Maisons-Alfort 23, avenue du Général de Gaulle 94706 Maisons-Alfort France; 6 Parasitologie comparée, UMR 7205 CNRS, Muséum National d’HistoireNaturelle CP52 61, rue Buffon 75231 Paris Cedex 05 France

**Keywords:** *Trichosomoides*, *Trichinella*, rodent, intramuscular development, *Trichosomoides*, *Trichinella*, rongeur, développement intramusculaire

## Abstract

*Trichosomoides nasalis* (Trichinelloidea) is a parasite of *Arvicanthis niloticus* (Muridae) in Senegal. Female worms that harbour dwarf males in their uteri, occur in the epithelium of the nasal mucosa. Young laboratory-bred *A. niloticus* were either fed females containing larvated eggs or intraperitoneally injected with motile first-stage larvae recovered from female uteri. Both resulted in successful infection. Organs examined during rodent necropsy were blood and lymphatic circulatory systems (heart, large vessels, lymphnodes), lungs, liver, kidneys, thoracic and abdominal cavities, thoracic and abdominal muscular walls, diaphragm, tongue, and nasal mucosa. Development to adult nasal stages took three weeks. Recovery of newly hatched larvae from the peritoneal fluid at four-eight hours after oral infection suggests a direct passage from the stomach or intestinal wall to the musculature. However, dissemination through the blood, as observed with *Trichinella spiralis*, cannot be excluded even though newly hatched larvae of *T. nasalis* are twice as thick (15 μm). Developing larvae were found in histological sections of the striated muscle of the abdominal and thoracic walls, and larvae in fourth moult were dissected from these sites. Adult females were found in the deep nasal mucosa where mating occurred prior to worms settling in the nasal epithelium. The present study shows a remarkable similarity between *T. nasalis* and *Trichinella* species regarding muscle tropism, but the development of *T. nasalis* is not arrested at the late first-larval stage and does not induce transformation of infected fibres into nurse cells. *T. nasalis* seems a potential model to study molecular relations between trichinelloid larvae and infected muscle fibres.

## Abstract (fr)

*Trichosomoides nasalis* (Trichinelloidea) est parasite d’*Arvicanthis niloticus* (Muridae) au Sénégal. Les vers femelles, qui contiennent des mâles nains intra-utérins, résident dans l’épithélium de la muqueuse nasale. De jeunes *A. niloticus* d’élevage ont ingéré des femelles avec des oeufs larvés, ou ils ont été inoculés par voie intrapéritonéale avec des larves écloses de stade 1, mobiles, extraites des utérus des femelles. Les rongeurs se sont parasités avec les deux protocoles. Les organes examinés à l’autopsie des rongeurs ont été les systèmes sanguin et lymphatique (coeur, gros vaisseaux, ganglions lymphatiques), les poumons, le foie, les reins, les cavités thoracique et abdominale, les parois musculaires thoracique et abdominale, le diaphragme, la langue et la muqueuse nasale. Le développement jusqu’au stade adulte s’effectue en trois semaines. La récolte de larves nouveau-nées dans le liquide péritonéal quatre-huit heures après l’infection orale suggère un passage direct de la paroi digestive vers la musculature. Cependant, une dissémination par le sang, telle qu’elle est observée avec *Trichinella spiralis*, ne peut être exclue bien que les larves à l’éclosion de *T. nasalis* soient deux fois plus épaisses (15 μm). Les larves en développement ont été trouvées dans les parois musculaires abdominale et thoracique et elles ont été identifiées sur coupes histologiques dans les fibres musculaires. Les larves en mue 4 ont été récoltées dans l’abdomen et le thorax, ainsi que, pour les femelles seulement, dans la partie profonde de la muqueuse nasale où l’accouplement a lieu, avant l’installation des femelles dans l’épithélium nasal. Cette étude met en évidence une remarquable similitude entre *T. nasalis* et les espèces de *Trichinella* – le tropisme musculaire –, cependant, le développement de *T. nasalis* ne s’arrête pas au stade 1 âgé et il n’induit pas la transformation des fibres infectées en cellules-nourricières. *T. nasalis* pourrait être un modèle intéressant pour étudier les mécanismes moléculaires des relations entre les larves de trichinelloïdes et les fibres musculaires infectées.

## Introduction

The African Grass Rat *Arvicanthis niloticus* (Geoffroy) is a species of rodent in the family Muridae. It is mainly distributed in the Sahel and the sudano-zambesian Savanna belt, which includes Senegal. Its natural habitats are dry savanna, moist savanna, subtropical or tropical moist shrubland, arable land, pasture land, rural gardens, urban areas, irrigated land and seasonally flooded agricultural land. However, little is known about its biology and actual occurrence (Musser & Carleton, 2005).

The trichosomoidin genera *Trichosomoides* Railliet, 1895 and *Anatrichosoma* Smith & Chitwood, 1954, are rarely recorded trichinelloids. They are primarily parasites of the epithelial layer of the mucosa of various organs (Anderson, 2000). Adults of some invade the digestive tract, similar to the related and extensively studied *Trichinella spiralis* (Owen, 1835) (*cf*. Gardiner, 1976), others parasitise the skin, buccal cavity, cornea and sclera, nasal cavities and passages, or the bladder of their hosts. *Trichosomoides* spp. inhabit the latter two organs (Antonakopoulos *et al.*, 1991; Serakides *et al.*, 2001; Diagne *et al.*, 2000 & 2004).

Male *Trichosomoides* are dwarfs and live in the uteri of the females. Larvated eggs are laid and expelled with the faeces, urine or nasal mucus. Hosts become infected when ingesting these eggs. To date, development in the host has only been investigated in *T. crassicauda* (Bellingham, 1840), a parasite of the bladder of rodents (Yokogawa, 1921; Thomas, 1924). Findings of Diagne *et al*. (2000) that *Trichosomoides nasalis* Biocca & Aurizi, 1961 occurs frequently in the nasal cavities of *A. niloticus* in Senegal, encouraged further studies on the biology of this genus.

A previous study on the pathological effects of *T. nasalis* at various sites in its host had raised two questions (Diagne *et al.*, 2004). The first concerned the migration route of *T. nasalis* from the digestive tract to the nasal mucosa. In this context, their finding of sections of two female worms in the connective tissue of the external aspect of the upper maxillae, suggested tissue migration. The second question addressed their mating behaviour. Diagne *et al*. (2000) reported that by far the majority of nasal females contained males, and free males were the exception. However, when and in what tissue or cavity the males entered the females had not been established. In *T. crassicauda*, copulation takes place in various regions of the urogenital tract of rats (Thomas, 1924). Further information can be gleaned from species of the closely related genus *Anatrichosoma*. Here, males and females are of similar size, and males do not invade females permanently. Little & Orihel (1972) observed a mating of *Anatrichosoma buccalis* Pence & Little, 1972 in the multilayered epithelium of the tongue of opossums: the posterior half of the male had been inserted into the genital tract of the female, but was subsequently retracted. The authors did not comment on the predilection site of males between matings. In *Anatrichosoma cynamolgi* Smith & Chitwood, 1954, a male was identified in the connective tissue underlying the epithelium of the nasal passages of a monkey, where it occupied the lumen of a, possibly lymphatic, vessel (Long *et al.*, 1976). In contrast, *Anatrichosoma haycocki* Spratt, 1982, copulates in the lumen of the paracloacal glands of dasyurids (Spratt, 1982).

The aims of the present study were to determine the migration route of *T. nasalis* from the intestinal lumen to the nasal mucosa, to identify the mating site, to describe the developmental stages and to establish the site of larval growth. One of the major findings reported herein is that larval development occurs in the musculature of the abdominal and thoracic walls. Thus, *T. nasalis* shows remarkable similarities with species of *Trichinella* Railliet, 1895, in particular non-encapsulated ones, since they do not induce the profound cellular remodelling associated with nurse cell formation (Purkerson & Despommier, 1974; Ko *et al.*, 1994; Despommier, 1998; Bruschi *et al.*, 2009).

## Materials and Methods

### Breeding and maintenance of *A. niloticus*

*Arvicanthis niloticus* were trapped in the field as described previously (Diagne *et al.*, 2000). They were captured near Dakar in the Niayes (14° 50’ 270’’ N, 17° 12’ 360’’ W) and in the region of Saint Louis, at Savoigne (16° 09’ 247’’ N, 16° 18’ 225’’ W). These animals were used to establish an infection-free breeding colony at the animal facilities of the Université Cheikh Anta Diop of Dakar, which provided all the young rodents used in this study. The experimental animals were kept in groups of 2-6 in transparent polycarbonate cages with stainless steel lids and a floor area of 549 cm^2^. Sterilized water was freely available in 100 ml or 500 ml propylene water bottles with stainless steel spouts and animals had unlimited access to a commercially available diet (Les Moulins Sentenac, Dakar, Senegal).

### Experimental infection of *A. niloticus*

Adult *T. nasalis* were obtained from either naturally or experimentally infected *A. niloticus*. Rodents were killed by cervical dislocation, and gravid female worms were collected from the nasal mucosa in accordance with earlier studies by Diagne *et al*. (2000). Two- to four-week-old *A. niloticus* were infected experimentally by one of two routes. The oral route was used in rodents of the first series of experiments (*n* = 102). Rodents were given no food or water for 24 h, and subsequently fed 1-4 freshly recovered females containing mature eggs. For this purpose, females were suspended in a drop of saline at the tip of a syringe (without needle) that the rodents were allowed to lick off. Any remaining liquid was gently squirted into the back of the buccal cavity. During the study a few embryonated eggs were noted in the lungs and the protocol was refined. The fluid was more gently and slowly pushed into the mouth and after using this method no more eggs were found in the lungs. Since the infection of rodents with intact females had proven unsuccessful, each female was first perforated, using a needle under a stereomicroscope. This seemed to facilitate the release of larvated eggs and some larvae hatched from the eggs while still in the female. The second series of rodents (*n* = 52) were infected intraperitoneally on the ventral line in the anterior part of the abdomen. Each was injected with highly motile, newly hatched larvae recovered from mature females of *T. nasalis*. Approximately 50 motile larvae were obtained from each female. The exact number of larvae administered was not determined and varied from a few to 35, and a few larvated eggs might have been mixed in with the free larvae. A small series of rodents were re-infected orally at ten day intervals and worms recovered from these were used for morphological studies only.

### Dissection of experimentally infected *A. niloticus*

Experimentally infected hosts were killed with chloroform to avoid bleeding. The skin was removed and discarded to eliminate possible contamination with phoretic nematodes that could interfere with the recovery of *T. nasalis* larvae at the early stage of development. The thoracic and abdominal cavities were opened separately and each briefly immersed in saline to recover any worms (corresponding figures in Table I in italics). The blood vessels of the heart were ligated, and blood was recovered from the right heart cavities. The lungs and trachea were teased apart in saline. In the abdominal cavity, the aorta, *vena cava* and collateral vessels were ligated, isolated and their blood examined. Following the example of previous studies on filarial nematode migration (Maréchal *et al.*, 1996; Wanji *et al.*, 1990), the main abdominal lymph nodes (mesenteric nodes and lumbar-iliac chain) as well as the cervical nodes were removed and dissected. The mesentery, digestive tract, liver and kidneys were separated and teased apart. Initially, the thoracic and abdominal muscles were lacerated only grossly (corresponding figures in Table I in normal text). Later, to improve recovery rates, an effort was made to finely dissect the thoracic and abdominal muscles, as well as the diaphragm and tongue (corresponding figures in Table I in bold). The nasal mucosa was dissected from three days post-infection (dpi) onwards.

**Table. I.** Localization of *Trichosomoides nasalis* in orally infected *Arvicanthis niloticus* (*n* = 67).

Time postinfection a	Infected/dissected hosts	Stomach	Lungs	Abdomen c	Total no. of worms per host
45 mpi	0/1	0	0	**0**	0
60 mpi	1/2	0	2/0	**0**	2
90 mpi	1/1	3*	5	**0**	8
2 hpi	3/3	4*/0/2*	19/1/0	**0**	23/1/2
	1/2	1*/0	0/0	**0**	1/0
4 hpi	1/1	0	4	1	5
6 hpi	1/1	0	1*	*13*	14
8 hpi	1/2	0/0	0/0	0/**12** + *6*	0/18
2 dpi	0/1	0	0	**0**	0
3 dpi	1/1	0	0	5	5
	0/1	0	0	**0**	0
4 dpi	2/2	0	0/1*	10/ 0	10/1
	1/1	0	0	**8**	8
	0/1	0	0	**0**	0
5 dpi	1/1	0	1	4	5
	0/1	0	0	**0**	0
6 dpi	0/2	0	0	0	0
7 dpi	3/7	0	6*/3/1	0/0/0	6/3/1
	1/1	0	0	**19**	19
8 dpi	1/4	0	10*	3	13
	1/1	0	0*	**5**	5
	0/1	0	0	**0**	0
9 dpi	1/6	0	2	0	2
	1/2	0	0	**2**	2
10 dpi	1/6	0	0	1	1
12 dpi	0/2	nd b	0	0	0
	1/1	nd	0	**5**	5
13 dpi	0/1	nd	0	**0**	0
	1/1	nd	nd	**4**	4
14 dpi	1/1	nd	nd	**2**	2
	1/1	nd	nd	**3**	3
	1/1	nd	nd	**1**	1
15 dpi	0/1	nd	0	**0**	0
	1/1	nd	nd	**0**	0
16 dpi	1/3	nd	nd	**2**	2
17 dpi	1/2	nd	nd	**2**	2
Total	31/67	10	56	108	174

a dpi: days post-infection; hpi: hours post-infection; mpi: minutes post-infection.

b nd: not determined.

c worms recovered from grossly lacerated wall: normal text; finely dissected wall: bold; abdominal cavity: italics.

* in addition to undeveloped larvae, larvated eggs were present as well.

All blood, organ or tissue samples were placed into separate petri dishes containing saline. Each was examined several times, starting one hour after they had been teased apart. Petri dishes were kept overnight at 4-7 °C and re-examined. Petri dishes containing blood were finally treated with 5 % acetic acid to lyse red blood cells and examined again. Developing small worms were fixed in 10 % formalin and adult large worms were fixed in hot 70 % ethanol. Gravid females, ≥ 40 days old, containing larvated eggs were not fixed, but used to infect naive *A. niloticus*.

Among the 102 orally infected rodents, 67 were dissected from 45 minutes post-infection (mpi) to 17 dpi following the complete protocol (see Table I for details), whereas only the nasal mucosa was dissected in 35 rodents examined 18-74 dpi. The 52 intraperitoneally infected hosts were dissected from 2 hours post-infection (hpi) to 32 dpi, and some of them were partly fixed for histology (Table II).

**Table. II.** Localization of *Trichosomoides nasalis* in intraperitoneally infected *Arvicanthis niloticus* (*n* = 52).

Time postinfection a	Infected/dissected hosts	Abdomen c	Thorax c	Nasal mucosa	Total no. of worms per host
2 hpi	1/1	**0** + *19*	**0**	nd d	19
4 hpi	1/1	**0** + *20*	**0**	nd	20
6 hpi	1/1	**2** + *33*	**0**	nd	35
8 hpi	1/1	**13** + *0*	**0**	nd	13
	1/1	**7** + *20*	**0**	nd	27
4 dpi	1/1	**4** + *0*	**0**	0	4
9 dpi	1/1	**5** + *0*	**0**	0	5
12 dpi	1/1	**6** + *0*	**0**	0	6
	2/5	**0/1/0/0/1**	**0**	0	0/1/0/0/1
14 dpi	4/4	**2/1/1/1**	**0/2/0/0**	0	2/3/1/1
14 dpi histo b	1/3	1 histo	**0**	0 histo	1
15 dpi	1/1	**2**	**0**	0	2
16 dpi	1/2	**0/0**	**0/1**	0	0/1
17 dpi	4/4	**0/0/0/3**	**4/2/1/0**	0	4/2/1/3
17 dpi histo b	1/4	1 histo	**0**	0 histo	1
18 dpi	1/1	**0**	**1**	0	1
18 dpi histo b	0/4	0 histo	**0**	0 histo	0
19 dpi histo b	4/4	0 histo	**1/1/2/2**	0 histo	1/1/2/2
20 dpi	3/5	**0**	**0/0/0/3/0**	1/0/0/0/2	1/0/0/3/2
21 dpi	2/3	**0**	**0**	0/5/4	0/5/4
30 dpi	2/2	**0**	**0**	3/3	3/3
32 dpi	2/2	**0**	**0**	5/3	5/3
Total	36/52	143	20	26	189

a dpi: days post-infection; hpi: hours post-infection.

b histo: the abdominal musculature and nasal mucosa of these hosts were used for histological sections and worms were recorded from these.

c worms recovered from grossly lacerated wall: normal text; finely dissected wall: bold; abdominal cavity: italics.

d nd: not determined.

### Morphological analysis of worms

The majority of worms were cleared in lactophenol and drawn under a compound microscope equipped with a camera lucida. Their length and width were measured on drawings as described in Diagne *et al*. (2000). Detailed studies were performed on several worms recovered from single infections as well as re-infected rodents, and the following developmental stages were distinguished: larvae, moulting unsexed larvae, fourth-stage females, males and females in fourth moult and adults. Unless otherwise stated, measurements are in micrometres.

### Histology

Intraperitoneally infected rodents were sacrificed from 14-19 dpi, and the abdominal wall or head or both fixed *in toto* in 10 % formalin (Table II). After dissection, samples of the muscles of the abdominal and thoracic walls, and the entire snout were prepared as described in Diagne *et al*. (2004), embbeded in paraffin wax, sectioned at 5 μm thickness, and stained with Mayer’s haemalum and eosin.

## Results

### Prevalence and mean intensity of infection

Both infection routes were successful as shown by dissections of the nasal mucosa on or later than 30 dpi. With the oral route, the prevalence of *T. nasalis* was 100 % (*n* = 8 rodents; from 33-74 dpi). The mean intensity of infection was 5 (range: 1-15), based on the number of females, as nearly all males had invaded the uteri by then. From 18-25 dpi, the prevalence in the nasal mucosa was 29.6 % (*n* = 27 rodents), suggesting that a proportion of worms were still migrating in tissues three weeks after oral infection. From 45 mpi to 17 dpi, the prevalence was 46.3 % (*n* = 67), with a mean intensity of 6.1 (1-24), based on the sum of unsexed larvae, and male and female larvae. It was obvious that once the larvae had invaded the tissue, not all of them could be recovered, even after the dissection techniques had been improved.

With the intraperitoneal route of infection, the final prevalence was 100 % (*n* = 2 rodents; 30 and 32 dpi, respectively) and the mean intensity of infection, based on female worms, was 3.5 (3-5). From 4-18 dpi, the prevalence was 75 % (*n* = 28), and the mean intensity was 5.1 (1-6).

### *Trichosomoides nasalis* localizations

After oral infection, from 45 mpi to 17 dpi the entire body of the host was examined for migrating worms. Newly hatched larvae and larvated eggs could be found in the oesophagus and stomach up to 2 hpi, but no later than that. Among the numerous organs and tissues examined, only a few contained worms. Indeed, the mesenteric vessels, collateral vessels, large blood vessels (aorta, vena cava), lymph nodes, kidneys, intestine, spleen and tongue were never found to be infected and were therefore omitted from Table I. The following organs or tissues were rarely infected and are also excluded from Table I: one of 52 hosts harboured a single larva in the oesophagus at 2 hpi, while one larva each were found in the heart and liver of one of 57 hosts at 5 dpi; the diaphragm of one of 49 and the thoracic muscular wall of one of 67 hosts were infected with one larva each, 2 hpi and 15 dpi, respectively, and the nasal mucosa was first infected at 17 dpi, and one of 54 rodents harboured 10 worms. The remaining organs and tissues were infected more regularly (Table I). One, 13 and six larvae were recovered from the abdominal cavity of a single rodent each at 4, 6 and 8 hpi, respectively (corresponding figures in Table I in italics). The lungs of 13 of 45 (28.9 %) rodents examined from 1 hpi to 9 dpi harboured 1-19 larvae per host. In addition, larvated eggs were present in the lungs of five of these hosts. The muscular abdominal wall of 17 of 56 (30.4 %) rodents examined from 8 hpi to 17 dpi were parasitised by 1-19 larvae per host.

In intraperitoneally infected rodents (Table II), larvae were absent from the following organs and tissues: the spleen, lymphatic nodes, chambers of the heart, lungs, liver, diaphragm and tongue. These organs are thus not represented in Table II. From 2-8 hpi, 81 % of the recovered larvae were found when opening the abdominal cavity in saline. The earliest that larvae were detected in the abdominal wall was 6 hpi, and by 4 dpi all larvae had invaded the abdominal wall. From 4-20 dpi, worms were found in the abdominal and/or thoracic wall. Worms were first found in the nasal mucosa at 20 dpi.

### *Trichosomoides nasalis* growth and development

**Fig. 1.**
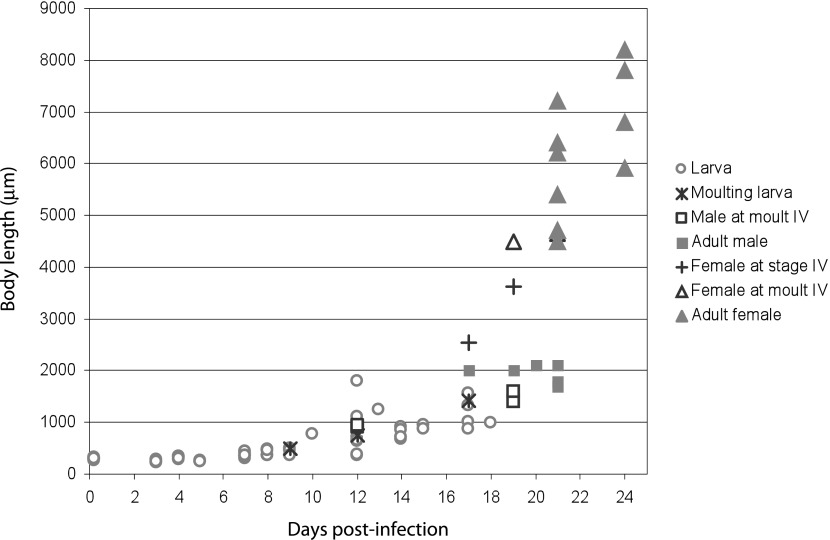
Growth and development of *Trichosomoides nasalis* in *Arvicanthis niloticus* within 24 days post-infection. Body-length and developmental stage of 93 worms recovered from six hours to 24 days postinfection are presented. Data from oral and intraperitoneal infection are pooled.

**Fig. 2.**
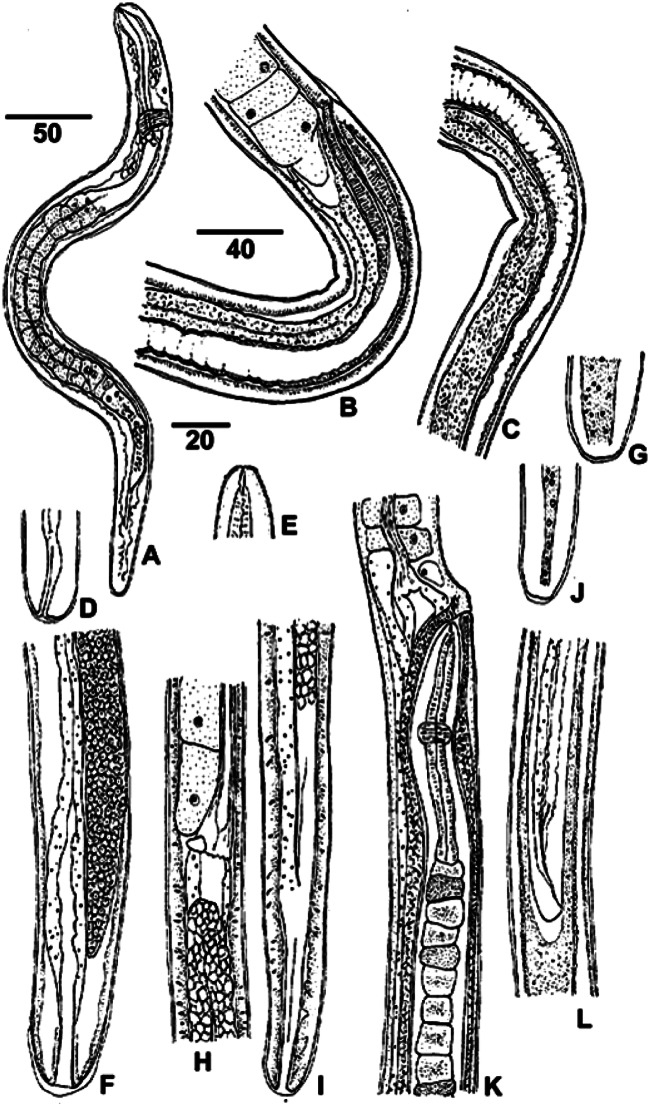
Morphology of developing *Trichosomoides nasalis* in *Arvicanthis niloticus*. A. First-stage larva, three days old, showing nerve ring, muscular oesophagus, long stichosome and short intestine, left lateral view. B-D. Female larva 2,800 μm long at very beginning of fourth moult; B. Oesophageal-intestinal junction and vulva (under exuvial sheath), vagina and distal part of uterus, right lateral view; C. Continuation of B, with uterus and intestine; D. Posterior extremity with rectum, lateral view. E-G. Female larva, 2,350 μm long; E. Head and stylet; F. Posterior region with ovary, intestine and rectum, and slight terminal exuvial sheath, dorso-ventral view; G. Bacillary band at posterior extremity, lateral view. H. Adult male 1,750 μm long, oesophageal-intestinal junction 1,050 μm from head. I. Same male, posterior extremity with intestine (on left), ejaculatory duct and cloaca, lateral view. J. Bacillary band at posterior extremity of a male, lateral view. K-L. Adult female 5,000 μm long with male; K. Male anterior part, its head oriented to vulva (note the stichocytes dark or clear); L. Male posterior part in uterus. A-G: worms from abdominal wall, H-L: worms from nasal mucosa. All worms recovered from series of re-infected rodents, except the first-stage larva. Scale bars in μm: A, K, L, 50; B, C, 40; D-J, 20.

**Fig. 3.**
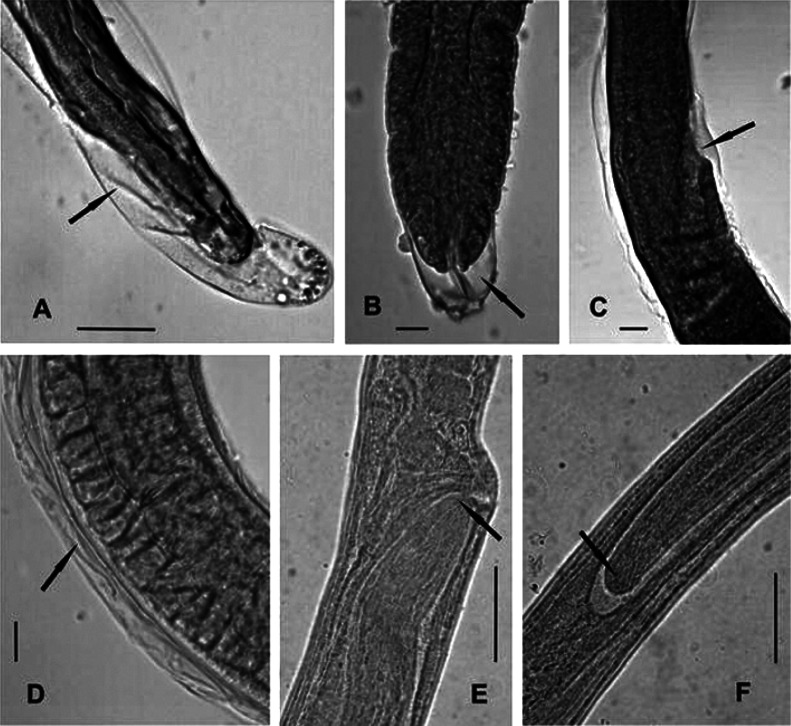
Moulting *Trichosomoides nasalis* and young adults from *Arvicanthis niloticus*. A. Moulting, unsexed larva, 450 μm long, at 9 days post-infection (dpi). B-D. Three females in fourth moult, 3,200, 3,700 and 4,400 μm long, at 17 dpi; B. Posterior extremity with exuvium and the cuticular lining of the rectum; C. Vulvar region (arrow), right lateral view; D. Body exuvium. E-F. Female 5,000 μm long with intra-uterine male; E. Male anterior part close to vulva; F. Male posterior part. Scale bars in μm: A, 25; B, C, D, 20; E, F, 40. A and B: worm from abdominal muscular wall, C-F: from nasal mucosa.

Larvae extracted from eggs (*n* = 8; 6 hpi; from abdominal residues) were 288.7 (250-310) long and 15.3 (12-18) wide. No differences in worm growth were apparent, irrespective of the infection route used. Therefore data from oral and intraperitoneal infections were pooled. Lengths of 93 worms, recovered from 6 hpi to 24 dpi, were measured (Fig. 1). Until 9 dpi, larvae grew slowly, approximately doubling their length, not exceeding 0.5 mm. Thereafter, growth was significant. Both sexed and unsexed larvae were seen moulting (Figs 1-3). Data on moulting dates and detailed worm measurements from intraperitoneal infections are presented in Table III. Larvae of increasing size (490, 750, 1,425) were moulting at 9, 12 and 17 dpi, respectively. Males in fourth moult were about 1,500 long at 19 dpi. Two females in fourth moult (vulva closed, genital tract developed) were 4,500 and 4,650 long at 19 and 21 dpi, respectively. The first adult male was found at 17 dpi and was 2,000 long. The first adult female was found at 21 dpi and was 4,700 long. Variations in the development of the worms in time and size are presented in Fig. 1. Several moulting worms of different sizes were also recovered from re-infected rodents. They could not be dated but were used for morphological analysis (Fig. 2).

**Table III.** Measurements of *Trichosomoides nasalis* at different stages related to time and localization in orally infected *Arvicanthis niloticus*. All measurements in micrometres.

Stage	Body length	Width at mid-body	Total oesophagus length	Muscular oesophagus length	Apex to vulva	Days post-infection	Localization
Larva^a^	208	18	140	60	–	3	abdominal
Moulting larva	490	25	275	90	–	9	abdominal
Moulting larva	750	30	380	120	–	12	abdominal
Moulting larva	1,425	25	820	165	–	17	abdominal
Male at 4^th^ moult	1,400	40	780	150	–	19	thoracic
Male at 4^th^ moult	1,600	40	900	140	–	19	thoracic
Adult male	2,000	35	1,170	155	–	17	abdominal
Adult male	2,000	40	1,100	160	–	19	thoracic
Adult male	2,100	40	1,100	160	–	20	nasal
4^th^ stage female	2,550	50	1,170	215	1,150	17	thoracic
4^th^ stage female	3,625	45	1,450	225	1,500	19	thoracic
Female at 4^th^ moult	4,500	50	1,500	200	1,550	19	thoracic
Female at 4^th^ moult	4,650	60	1,700	200	1,750	21	nasal
Adult female	4,700	60	1,650	210	1,700	21	nasal
Adult female	5,400	70	1,720	225	1,800	21	nasal
Adult female	6,400	90	1,800	225	1,850	21	nasal

a from intraperitoneal infection.

The buccal stylet and bacillary bands were present in all larvae and adults studied (Fig. 2D, G, J). At 3 dpi, the oesophagus extended into the anterior 2/3 of the body, and the stichosome could be seen in the posterior half of the oesophagus. The intestine was short (Fig. 2A). During worm growth, the ratio oesophagus/ body length was reduced to 1/2 in the male and 1/3 in the female. In both sexes, dark stichocytes alternated more or less regularly with clear stichocytes (Fig. 2K). Sexes could be distinguished when larvae were ≥ 1 mm long, male larvae being thinner than female larvae and the male cloaca being twice as long as the female rectum (75 and 30 long, respectively; Fig. 2). Adult worm morphology was similar to that described previously (Diagne *et al.*, 2000), and the length of five females, collected at 32 dpi, ranged from 9,990 to 13,600.

### Organs containing developing and adult *T. nasalis*

In the lungs, no growth was apparent. First-stage larvae, at 9 dpi, did not exceed 320 in length, contrary to those from the abdominal wall which had reached a length of 490. Larvated eggs were also recovered from the lungs of five hosts after oral infection. In the heart and diaphragm, two first-stage larvae, found no later than 5 dpi, did not show any sign of development.

Following both routes of infection, developing larvae were found in the musculature of the abdominal wall and, at a lower frequency, in the thoracic wall (Tables I & II). Worms were unsexed young larvae, fourth-stage females, males in fourth moult and three of a total of seven females found in fourth moult. A few adult males, but no adult females, were found at these sites as well.

The nasal mucosa contained the remaining four females in fourth moult (17-23 dpi; Table III). Notably, these moulting females, together with young males, were extracted from the deep layer of the mucosa. At later collection times, mature females containing one to two dwarf males were recovered from the upper epithelial layer whereas males alone were rare.

### *Trichosomoides nasalis* in striated muscle fibres

**Fig. 4.**
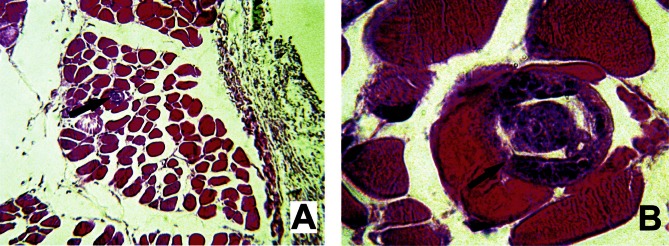
Fourth-stage larva of *Trichosomoides nasalis* in striated muscle fibre of *Arvicanthis niloticus*. A & B. Two magnifications of a section of abdominal wall fixed seventeen days following intraperitoneal infection and stained with Mayer’s Haemalum and Eosin. Intestine and gonad of *T. nasalis* are identified. Note that no collagen capsule and no inflammatory cells were observed around *T. nasalis* within the muscle tissue. Diameter of the larva: 33 μm.

The abdominal wall and nasal mucosa of 15 rodents had been prepared for histology (Table II). In sections of the abdominal wall of two of these, a single worm each was seen at 14 and 17 dpi, respectively. Both larvae were identified inside a muscle fibre (Fig. 4A). No collagen capsule or inflammatory cells were seen around the infected fibre. In fact, inflammation was limited to the internal aspect of the peritoneum, surrounding the area of inoculation. No major changes were noted in the infected fibre, but the myofibril structure seemed less defined (Fig. 4B). The worms identified at 14 and 17 dpi were of similar width (30 and 33, respectively) and were in the fourth stage.

## Discussion

The present study demonstrates that *T. nasalis* females, containing uterine males, settle in the nasal mucosa within three weeks of infection. The initial development takes place in the musculature of the abdomen and thorax. The migration routes, especially at the start and end of development, as well as the main features of development seen in this study are discussed here and compared with other nematodes, mainly the Trichinelloidea.

### Early and late migrations

The investigation of larval migration routes is challenging at best, and for a given species different authors may come to controversial conclusions (Yokogawa, 1921; Thomas, 1924). In *T. spiralis*, tiny larvae, 111- 125 long and 7 wide (Ali khan, 1966), are born at an early embryonic stage and disperse in the blood and lymphatic system (review in Anderson, 2000). The way that first-stage larvae of *T. nasalis* pass from the digestive tract to the abdominal musculature has not been fully revealed yet, but, at present, there is no strong evidence of blood or lymphatic migration. On the contrary, several observations made during the current study suggest direct tissue migration from the wall of the digestive tract. Firstly, despite thorough dissection of the hosts, no larvae were recovered from blood vessels or the heart from 45 mpi to 8 hpi (a single cardiac larva was recovered at 5 dpi). In contrast with filarial species (Wanji *et al.*, 1990; Maréchal *et al.*, 1996), no larvae were recovered from the lymph nodes. Secondly, the few *T. nasalis* recovered from the lungs did not seem to be the result of blood dissemination. Because eggs were present in the lungs as well, it would appear that the parasites had accidentally been aspirated when the liquid used for oral infections was squirted into the back of the buccal cavity of the young rodents. The same technique for oral infections was subsequently refined and lungs were no longer found to be infected. Thirdly, in orally infected hosts examined at an early stage after infection (2-8 hpi), newly hatched larvae were found in the abdominal cavity of three rodents (one, 13 and six larvae, respectively; Table I). In intraperitoneally infected hosts, larvae were seen in the abdominal wall of three rodents as early as 6-8 hpi (Table II) and no larvae were recovered from blood or lymphatic vessels during this period. We, therefore, suggest that first-stage larvae of *T. nasalis* progress from the lumen of the digestive tract to muscle fibres through tissue migration, using their buccal stylet and secretions from the stichosome. As observed by Thomas (1924) with *T. crassicauda*, the movements of the stylet are very active in larvae from the abdominal cavity.

Upon completion of their development in the musculature, advanced female fourth-stage larvae and young males of *T. nasalis* migrate to the nasal mucosa by an as yet unknown route. A lymphatic migration cannot be excluded, because the Pecquet cysterna in the abdominal region is close by. Alternatively, the stylet of *T. nasalis*, still present in the late stages, may help penetrating the mucosa in conjunction with worm secretions. For *Trichinella* species, which have no stylet at the late infective first stage, it has been shown that excretory/secretory (ES) antigens composed of tyvelose-bearing glycoproteins, play an important role in the penetration of epithelial intestinal cells (ManWarren *et al.*, 1997; Appleton, 2001; Appleton & Romaris, 2001). Indeed, serine proteases, which are abundant within *Trichinella*’s ES antigens could have an important function in the host-invasion process (Dzik, 2006), and facilitate migration within tissues.

In *T. nasalis*, mating is linked to late tissue migration. It seems to occur in the deeper layers of the nasal mucosa. Since females in the fourth moult, as well as some males, were recovered from the connective layer of the mucosa, we assume that mating occurs at this site, prior to females reaching the nasal epithelium. This is similar to the mating behaviour of *A. cynamolgi*. In the few histological studies concerning this species, males were found in the nasal submucosa of infected Rhesus monkeys, *Macaca mulatta* (Zimmermann), but not in the epithelial mucosa (Long *et al.*, 1976). This contrasts with the mating behaviour of two other Trichosomoidinae, described as occuring in the lumen of the urogenital tract of the rat for *T. crassicauda* (Thomas, 1924), and the paracloacal glands of *Antechinus* spp. for *A. haycocki* (Spratt, 1982). The possibility of the latter being a false result cannot be ruled out, as delicate epithelia are easily ruptured during dissection.

### Development in muscle fibres

The development of *T. nasalis* larvae to the adult stage takes three weeks, similar to that of *T. crassicauda* (Thomas, 1924). With *T. nasalis*, larvae were seen moulting at different lengths and at different times throughout the experimental period (Table III). At least three moults were identified. A moult observed at 9 dpi (Fig. 3A) was likely the first moult since the larva was only 490 long. The second moult was likely seen at 12 dpi, in a larva that was 750 long. The fourth moult occurred when worms could be identified as males or females. In total, 20 moulting larvae were recovered from all experimentally infected rodents, including larvae of intermediary size, which might represent the second or third moult. (Kozek, 1971a, 1971b) demonstrated that in *T. spiralis*, similar to other groups of nematodes, the classic number of four moults was the rule. However, these moults occurred within a very short period, from 9-28 hpi. The infective, late first-stage larvae of *T. spiralis* have grown in modified nurse cells and are thus very different from the infective, newly hatched larvae of *T. nasalis*. As a consequence, the timing of moults in *T. nasalis* is not as highly specialized as in *T. spiralis*.

Developing worms of *T. nasalis* were recovered regularly from the musculature of the abdominal wall and, at a lower frequency, the thoracic wall (Tables I & II). In addition, developing larvae were found enclosed within muscle fibres (Fig. 4). This specific localization is a first record for Trichosomoidinae, the development of which has only been analysed for two species, *T. crassicauda* and *A. haycocki*. It may be a particular biological trait in *T. nasalis*, related to the nasal localization of adults, but we suggest that it might have passed unnoticed in other studies because these muscles were not amongst the examined organs (Thomas, 1924; Spratt, 1982). Indeed, the analysis was restricted to the blood system, liver, lungs and the sites where adults settle, urinary tract and posterior digestive tract, respectively. The muscular localization is particularly interesting, and might have phylogenetic significance, as species of *Trichinella* are known to develop in muscle fibres as well. However, the similarity between *Trichinella* spp. and *T. nasalis* is not complete. Firstly, the intramuscular phase of *Trichinella* corresponds to an extended growing phase of the first stage (Despommier *et al.*, 1975), whereas in *T. nasalis*, all larval stages occur in the muscular wall. Moreover, the development of *T. nasalis* does not seem to induce transformation of the muscle fibre into a nurse cell, as described for *T. spiralis* (Despommier, 1998). Interestingly, no collagen capsule and no inflammatory cells were observed around *T. nasalis* within the muscle tissues. These results approach *T. nasalis* to non-encapsulated *Trichinella* species such as *T. pseudospiralis* Garkavi, 1972, since this species also does not induce formation of a collagen wall, and the surrounding inflammatory response remains limited (Hulinska *et al.*, 1985; Haehling *et al.*, 1995; Bruschi *et al.*, 2009). Hence, the murid parasite *T. nasalis* might be a new model that is well worth investigating.

## References

[R1] Alikhan Z. The postembryonic development of *Trichinella spiralis* with special references to ecdysis. Journal of Parasitology, 1966, 52, 248–259

[R2] Anderson R.C. Nematodes parasites of vertebrates. Their development and transmission. 2nd Edition. CABI Publishing, Willingford, UK, 2000, 650 p.

[R3] Antonakopoulos G.N., Turton J., Whitfield P. & Newman J. Host-parasite interface of the urinary bladder-inhabiting nematode *Trichosomoides crassicauda*: changes induced in the urothelium of infected rats. International Journal for Parasitology, 1991, 21, 187–193 1869353 10.1016/0020-7519(91)90009-v

[R4] Appleton J.A. New insights into the intestinal niche of *Trichinella spiralis*, *in*: Parasitic nematodes: molecular biology, biochemistry and immunology. Kennedy M.W. & Harnett W. (eds), CABI Publishing, Wallingford, UK, 2001, 103–120

[R5] Appleton J.A. & Romaris F. A pivotal role for glycans at the interface between *Trichinella spiralis* and its host. Veterinary Parasitology, 2001, 101, 249–260 11707300 10.1016/s0304-4017(01)00570-2

[R6] Bruschi F., Marucci G., Pozio E. & Masetti M. Evaluation of inflammatory responses against muscle larvae of different *Trichinella* species by an image analysis system. Veterinary Parasitology, 2009, 159, 258–262 19046814 10.1016/j.vetpar.2008.10.038

[R7] Despommier D.D. How does *Trichinella spiralis* make itself at home? Parasitology Today, 1998, 14 (8), 318–323 17040798 10.1016/s0169-4758(98)01287-3

[R8] Despommier D.D., Aron L. & Turgeon L. *Trichinella spiralis*: growth of the intracellular (muscle) larva. Experimental Parasitology, 1975, 37, 108–116 1116513 10.1016/0014-4894(75)90058-2

[R9] Diagne M., Diouf M., Lochouarn L. & Bain O. *Trichosomoides nasalis* Biocca & Aurizi, 1961 et *T. spratti* n. sp. (Nematoda : Trichinelloidea), parasites des fosses nasales de muridés. Parasite, 2000, 7, 215–220 11031758 10.1051/parasite/2000073215

[R10] Diagne M., Vuong P.N., Duplantier J.M., Ba K., Thirionlochouarn L., Attout T. & Bain O. Histological study of *Trichosomoides nasalis* (Nematoda: Trichinelloidea) in the murid *Arvicanthis niloticus*, with associated pathology. Parasite, 2004, 11, 351–358 15638135 10.1051/parasite/2004114351

[R11] Dzik J.M. Molecules released by helminth parasites involved in host colonization. Acta Biochimica Polonica, 2006, 53, 33–64 16410836

[R12] Gardiner C.H. Habitat and reproductive behavior of *Trichinella spiralis*. Journal of Parasitology, 1976, 62, 865–870 1003275

[R13] Haehling E., Niederkorn J.Y. & Stewat G.L. *Trichinella spiralis* and *Trichinella pseudospiralis* induce collagen synthesis by host fibroblasts *in vitro* and *in vivo*. International Journal for Parasitology, 1995, 25, 1393–1400 8719950 10.1016/0020-7519(95)00080-1

[R14] Hulinska D., Grimm M. & Shaikenov B. The feeding mechanism of intracellular muscle larvae *Trichinella nativa* Britov et Boev, 1972 and *T. pseudospiralis* Garkavi, 1972. Folia Parasitologica (Praha), 1985, 32, 61–66 3988160

[R15] Ko R.C., Fan L., Lee D.L. & Compton H. Changes in host muscles induced by excretory/secretory products of larval *Trichinella spiralis* and *Trichinella pseudospiralis*. Parasitology, 1994, 108, 195–205 8159465 10.1017/s0031182000068293

[R16] Kozek W.J. The molting pattern in *Trichinella spiralis*. I. A light microscope study. Journal of Parasitology, 1971a, 57, 1015–1028 5133876

[R17] Kozek W.J. The molting pattern in *Trichinella spiralis*. II. An electron microcopse study. Journal of Parasitology, 1971b, 57, 1028–1038 5133878

[R18] Little M.D. & Orihel T.C. The mating behavior of *Anatrichosoma* (Nematoda: Trichuroidea). Journal of Parasitology, 1972, 58, 1019–1020 5078588

[R19] Long G.G., Lichtenfels J.R. & Stookey J.L. *Anatrichosoma cynamolgi* (Nematoda: Trichinellida) in rhesus monkeys, *Macaca mulatta*. Journal of Parasitology, 1976, 62, 111–115 815529

[R20] Manwarren T., Gagliardo L., Geyer J., McVay C., Pearce-Kelling S. & Appleton J. Invasion of intestinal epithelia *in vitro* by the parasitic nematode *Trichinella spiralis*. Infection and Immunity, 1997, 65, 4806–4812 9353069 10.1128/iai.65.11.4806-4812.1997 PMC175690

[R21] Maréchal P., Le Goff L., Petit G., Diagne M., Taylor D.W. & Bain O. The fate of filaria *Litomosoides sigmodontis* in susceptible and naturally resistant mice. Parasite, 1996, 3, 25–31 8731760 10.1051/parasite/1996031025

[R22] Musser G.G. & Carleton M.D. Superfamily Muroidea, *in*: Mammal species of the world: a taxonomic and geographic reference. Wilson D.E. & Reeder D.M. (eds), Johns Hopkins University Press, Baltimore, 2005, 894–1531

[R23] Purkerson M. & Despommier D. Fine structure of the muscle phase of *Trichinella spiralis* in the mouse, *in*: Trichinellosis. Kim C.W. (ed.), Intext Publishers Group, New York, 1974, 7–23

[R24] Serakides R., Ribeiro A.F.C., Silva C.M., Santos R.L., Nunes V.A. & Nascimento E.F. Proliferative and inflammatory changes in the urinary bladder of female rats naturally infected with *Trichosomoides crassicauda*: reports of 48 cases. Arquivo Brasileiro de Medicina Veterinària e Zootecnia, 2001, 53, 1–5

[R25] Spratt D.M. *Anatrichosoma haycocki* sp. n. (Nematoda: Trichuridae) from the paracloacal glands of *Antechinus* spp., with notes on *Skrjabinocapillaria skarbilovitschi*. Annales de Parasitologie Humaine et Comparée, 1982, 57, 63–71 7081890 10.1051/parasite/1982571063

[R26] Thomas L.J. Studies on the life history of *Trichosomoides crassicauda* (Bellingham). Journal of Parasitology, 1924, 10, 105–140

[R27] Wanji S., Cabaret J., Gantier J.C., Bonnand N. & Bain O. The fate of the filaria *Monanema martini* in two rodent hosts: recovery rate, migration and localization. Annales de Parasitologie Humaine et Comparée, 1990, 65, 80–88 2221759 10.1051/parasite/1990652080

[R28] Yokogawa S. On the migratory course of *Trichosomoides crassicauda* (Bellingham) in the body of the final host. Journal of Parasitology, 1921, 7, 80–84

